# Characteristics of Indoor PM_2.5_ Concentration in Gers Using Coal Stoves in Ulaanbaatar, Mongolia

**DOI:** 10.3390/ijerph15112524

**Published:** 2018-11-12

**Authors:** Miyoung Lim, Sainnyambuu Myagmarchuluun, Hyunkyung Ban, Yunhyung Hwang, Chimedsuren Ochir, Delgerzul Lodoisamba, Kiyoung Lee

**Affiliations:** 1 Department of Environmental Health Sciences, Graduate School of Public Health, Seoul National University, Seoul 08826, Korea; me02me0@snu.ac.kr (M.L.); ban_hyun@hanmail.net (H.B.); hyh@snu.ac.kr (Y.H.); delgerzul@snu.ac.kr (D.L.); 2 Department of Environmental Health, School of Public Health, Mongolian National University of Medical Sciences, Ulaanbaatar 14210, Mongolia; myagmarchuluun@mnums.edu.mn (S.M.); chimedsuren@mnums.edu.mn (C.O.); 3 Institute of Health and Environment, Seoul National University, Seoul 08826, Korea

**Keywords:** PM_2.5_, indoor air quality, ger, indoor stove, coal combustion

## Abstract

Coal combustion in ger areas is the main source of ambient air pollution in Ulaanbaatar (Mongolia). This study determined the characteristics of indoor PM_2.5_ concentrations in gers using coal stoves during winter. The study population consisted of 60 gers in the Chingeltei district of Ulaanbaatar. The indoor particle number concentration (PNC) in each ger was measured using a Dylos DC1700 particle counter for 24 h in January and February 2016. The PNC by Dylos was converted into the mass concentration using a calibration equation developed using a collocated real-time light scattering monitor adjusted by gravimetric measurement. The average 24 h PM_2.5_ concentration was 203.9 ± 195.1 μg/m^3^ in gers with traditional stoves (*n* = 29) and 257.5 ± 204.4 μg/m^3^ in those with improved stoves (*n* = 31). In the daily profile, concentrations were lower at night, increased in the early morning, and peaked up to noon. The temperature in gers was slightly higher than that recommended in winter. Many development-assistance programs have supported the installation of improved energy-efficient stoves. Better control measures are needed to improve the indoor air quality of gers.

## 1. Introduction

A ger is a traditional Mongolian dwelling. It is a portable, circular, felt-covered, wood lattice-framed dwelling that is equipped with a stove for heating and cooking. In suburban areas of Ulaanbaatar, the capital and largest city in Mongolia, hundreds of thousands of low-income families live in gers without basic urban services. Around 95% of ger households in Ulaanbaatar use coal and firewood for heating and cooking [1]. Coal combustion in gers is the main source of air pollution in Ulaanbaatar [2]. There are more than 160,000 gers in Ulaanbaatar and each burns an average of 5 tons of coal and 3 m^3^ of wood per year [3].

Ulaanbaatar is one of the most polluted cities in the world, particularly in winter. The average ambient concentration of particulate matter with a diameter of less than 2.5 μm (PM_2.5_) in winter was reported to be 148 μg/m^3^ at a central government monitoring site and 250 μg/m^3^ in the ger areas of Ulaanbaatar [4]. The permissible PM_2.5_ level of the Mongolian ambient air quality standard (MNS 4585: 2007) is 50 μg/m^3^ for a 24-hour average and 25 μg/m^3^ for the annual average. The PM_2.5_ concentration in winter exceeded the national standard of Mongolia by more than 10-fold. High concentrations of PM_2.5_ were reported both in the ambient air and indoor environments in the ger areas of Ulaanbaatar. The geometric mean concentration of PM_2.5_ in a ger was reported to be 127.8 μg/m^3^, significantly higher than the level in a formal apartment [5]. The average concentrations of PM_2.5_ in gers during the daytime in winter ranged from 178.4 ± 152.7 to 208 ± 173 μg/m^3^ [6,7].

Prolonged exposure to indoor pollutants can threaten the health of vulnerable residents. In Ulaanbaatar, children living in gers are exposed to higher indoor concentrations of PM_2.5_ than children living in wooden houses [8]. Respiratory diseases and neurodevelopmental disorders in children of Mongolia have been linked to exposure to toxic chemicals from outdoor and indoor air pollution [9]. Exposure to PM_2.5_ in Ulaanbaatar is estimated to be responsible for 1400 attributable deaths annually and 40,000 attributable disability-adjusted life years [10].

The stoves used in gers have a closed combustion chamber and a chimney vented to the outdoors. These stoves have short stacks (2 m), so the emissions remain low in the atmosphere and close to people’s living quarters [11]. The U.S. Millennium Challenge Corporation funded a program that developed energy-efficient stoves to improve air quality through the Energy and Environment Project (EEP). The stove project implemented by the Millennium Challenge Account (MCA) selected four top-lit-updraft design (TLUD) stove models and encouraged the installation of these stoves in several districts in Ulaanbaatar. The four TLUD stoves were the Ulzii (Silver Mini), Khas (Silver Turbo), Dul (Royal Single), and Golomt (Royal Double) stoves, which are commonly referred as improved stoves. The Asian Development Bank also supported the use of TLUD stoves, which were expected to reduce total particulate matter emissions by 50–80% [12]. An MCA energy efficiency project reported that the subsidized, improved stoves had 65% lower PM_2.5_ emissions during typical usage than traditional stoves [13]. Nevertheless, the indoor PM concentrations in homes using the improved stoves were not significantly lower [14]. It was necessary to determine indoor PM_2.5_ concentration by stove type for effectiveness evaluation of the control measure. Indoor coal combustion through the stove in gers required special attention as one of the major sources of PM_2.5_ in Mongolia.

The purpose of this study was to determine the characteristics of indoor PM_2.5_ concentrations by measuring the 24 h profile of indoor PM_2.5_ concentration in gers using traditional and improved stoves. Concentrations were measured using a particle counter that was calibrated with a collocated real-time light scattering monitor adjusted by gravimetric measurement.

## 2. Materials and Methods

### 2.1. Study Area and Participants

The study was conducted in Ulaanbaatar, Mongolia. In January, the average temperature was about −24.6 °C (range −26.5 to −15.6 °C) [15]. Ulaanbaatar is divided into nine districts, each of which is subdivided into 121 sub-districts (khoroo). Our study area included the Bayanzürkh and Chingeltei districts, two of the districts that obtained financial support from MCA for the purchase of improved TLUD stoves. We recruited similar numbers of gers with traditional and improved stoves through a primary health center doctor.

### 2.2. Calibration of the PM Monitor

Indoor concentrations of particulate matter (PM) were measured simultaneously using a Dylos DC1700 (Dylos Corporation, Riverside, CA, USA) based on light-scattering technology and a MicroPEM™ nephelometer light-scattering sensor with integrated filter collection (RTI International, Research Triangle Park, NC, USA) in 24 gers in the 17th sub-district of Bayanzürkh district during the day from 15 to 19 January 2016. The Dylos was co-located with the MicroPEM for daytime on a table roughly 1.2 m from the ground, and measurement were conducted 6 h per day (approximately 10:00–17:00).

The Dylos DC1700 monitored PM from > 0.5 μm to 2.5 μm in aerodynamic diameter (small particles) and PM > 2.5 μm in aerodynamic diameter (large particles). The measurement interval was 1-min for Dylos. The MicroPEM used 780 nm infrared laser nephelometer monitoring and allowed gravimetric measurement, simultaneously. For zero calibration for the inlet of MicroPEM, High Efficiency Particulate Air (HEPA) filter and pre-calibrated TSI 4146 flowmeter (TSI Inc., Shoreview, MN, USA) were used. The air flow rate for calibration was 0.50 L/min. The particles were collected gravimetrically on 3.0 μm pore size polytetrafluoroethylene (PTFE) 25 mm filter (Zefon International, Ocala, FL, USA) placed in the MicroPEM filter cassette. The filters were conditioned in a dry container for 48 h before weighing, and measured using microbalance in a temperature and humidity controlled room. The gravimetric weight of PM_2.5_ collected on the MicroPEM filter was compared to reading of laser particulate counter, Dylos.

### 2.3. Design of Measurement

The indoor PM concentration was measured for 24 h in 60 gers in the 18th sub-district of Chingeltei district, including 29 gers with traditional stove and 31 gers with improved TLUD stove. The participating households were recruited by doctors at a primary health center. The monitoring was conducted from 27 January to 20 February 2016. On each sampling day, similar numbers of gers with traditional and improved stoves were assigned. The indoor concentrations of PM were measured with a Dylos DC1700. Temperature and relative humidity were measured with a HOBO UX100-003 data logger (Onset Computer, Pocasset, MA, USA). The accuracy of the temperature senor was ±0.21 °C from 0° to 50 °C and the measurement range was −20° to 70 °C. The accuracy of the relative humidity senor was ±3.5% from 25% to 85% including hysteresis at 25 °C and the measurement range was 15% to 95%. The measurement interval was 1-min for both Dylos and HOBO. We also administered a simple questionnaire survey to the residents regarding the types and amount of fuel used, frequency of fuel addition, number of meals cooked, number of family members, and indoor smoking status.

### 2.4. Data Analyses

Simultaneous measurements with the Dylos DC1700 and MicroPEM in 24 gers were used to calibrate the Dylos DC1700 readings. The mass-weighted concentrations of PM_2.5_ obtained with MicroPEM were used as true values. Linear regression between the Dylos and MicroPEM readings was performed for calibration. The calibration equation from the 24 gers was applied to the 24 h Dylos measurements in 60 gers. The differences in the indoor PM concentrations by stove type were compared using Mann–Whitney U test. The daily average of indoor/outdoor ratio (I/O ratio) was calculated by the outdoor PM_2.5_ concentrations of the nearest station of the 18th sub-district of Chingeltei district (NMB station) in Ulaanbaatar for the corresponding days were accessed from the OpenAQ Platform (http://openaq.org) and originate from Agaar Air Quality (http://aggar.mn source). Temporal profiles of the indoor concentrations were drawn and compared by stove type. IBM SPSS Statistics ver. 22 (IBM, Armonk, NY, USA) was used for the statistical analyses and graphs were drawn with SigmaPlot 10.0 (Systat Software, San Jose, CA, USA).

The temporal variation in the 24 h real-time concentration was analyzed using peak analyses [16]. We defined a peak as a concentration exceeding 100 μg/m^3^ with an increase greater than 35 μg/m^3^ in 1 min (*C_i_* > 100 μg/m^3^, where *i* = 1, 2, …, *n*, *n* is the total number of min, and *C_i_*–*C_i_*_−1_ > 35 μg/m^3^). When there was more than one peak within a 15 min period, the maximum concentration within the 15 min interval was selected as the peak. The use of 35 μg/m^3^ as the definition of a peak was determined by the distribution of the difference. The 98.2 percentile of the distribution was 35 μg/m^3^. One peak per 15 min was selected based on the assumption that multiple peaks within a short interval might be caused by the same source, such as the combustion of coal fuel. We defined the ‘peak occurrence rate’ as the number of peaks per day and the ‘peak magnitude’ as the concentration at the peak.

## 3. Results

### 3.1. Comparison of the Dylos DC1700 and MicroPEM Results

The 24 h average concentrations with the Dylos DC1700 and MicroPEM were compared using linear regression analyses. Of the 24 pairs of measurements, 20 pairs were available for the comparison. For four pairs, the sampling time of the filter measurement exceeded the Dylos reading. The calibration equation for the Dylos measurement was determined by a linear regression as follows:

PM_2.5_ mass concentration (μg/m^3^) = 1.354 × Dylos PNC (#/ft^3^) × 10^−4^, (1)

**Figure 1:**
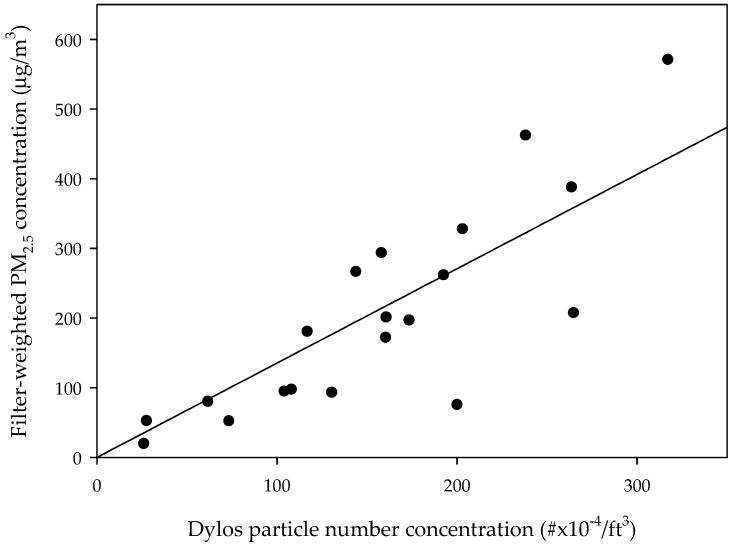
Comparison of the particle number concentration (#×10^−4^/ft^3^) determined using the Dylos DC1700 and the filter-weighted PM_2.5_ concentration (μg/m^3^) determined using the MicroPEM.

The Dylos PNC was the particle number concentration (PNC) obtained by subtracting the value for large particles from that for small particles. This equation was derived within the range 26–317 × 10^4^ particles/ft^3^ for the Dylos reading and 19–570 μg/m^3^ for the mass concentration (Figure 1).

### 3.2. Characteristics and Indoor PM_2.5_ Concentrations of 60 Gers

**Figure 2:**
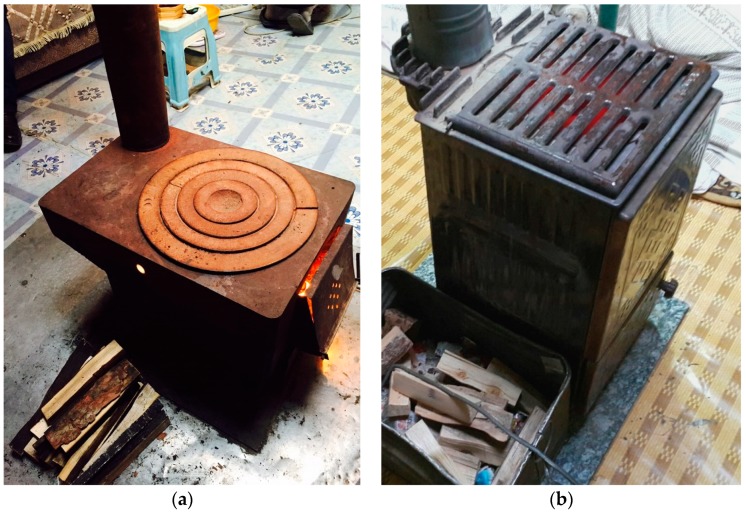
(**a**) A traditional Mongolian stove and (**b**) the Ulzii stove, one of the four improved top-lit-updraft design (TLUD) stoves supported by the Millennium Challenge Account (MCA).

Table 1 shows the characteristics of the 60 gers in which 24 h measurements were conducted. There were 29 gers with traditional stove (Figure 2a) and 31 gers with improved MCA stove (Figure 2b). Most of the stoves were fueled with coal, wood, and other materials. For households with traditional and improved stoves, the average fuel consumption was 13.1 ± 7.5 and 11.9 ± 5.6 kg per day, respectively, and the fuel-addition frequency was 3.5 ± 1.7 and 3.3 ± 1.9 times per day, respectively. The households cooked an average of 2.2 ± 1.4 times per day and contained an average of 4.0 ± 1.6 family members. Family members of seven gers smoked inside the ger. These factors did not differ statistically according to stove type. Indoor temperatures were 22.3 ± 8.9 °C in gers with traditional stove and 21.8 ± 8.1 °C in gers with improved stove. Relative humidity were 23.3 ± 8.8% in gers with traditional stove and 18.6 ± 5.1% in gers with improved stove (*p* < 0.001).

The indoor PM_2.5_ concentrations were very high. The average 24 h indoor concentration in gers was 236.1 ± 112.1 μg/m^3^ and the 30 min average concentration was as high as 797.2 μg/m^3^. The indoor concentrations were 203.9 ± 195.1 in gers with traditional stove and 257.5 ± 204.4 μg/m^3^ in gers with improved stove. The indoor PM_2.5_ concentrations were significantly higher in gers with improved stove than in gers with traditional stove (*p* < 0.001). The I/O ratios of 24 h average PM_2.5_ concentrations were 1.20 ± 0.68 (range 0.39–3.32) for gers with traditional stove and 1.50 ± 0.91 (range 0.30–3.77) for gers with improved stove. The I/O ratio was slightly higher in gers with improved stove; however, the difference was not statistically significant (*p* = 0.198).

### 3.3. The 24 h Profiles of PM_2.5_, Temperature, and Relative Humidity

**Figure 3:**
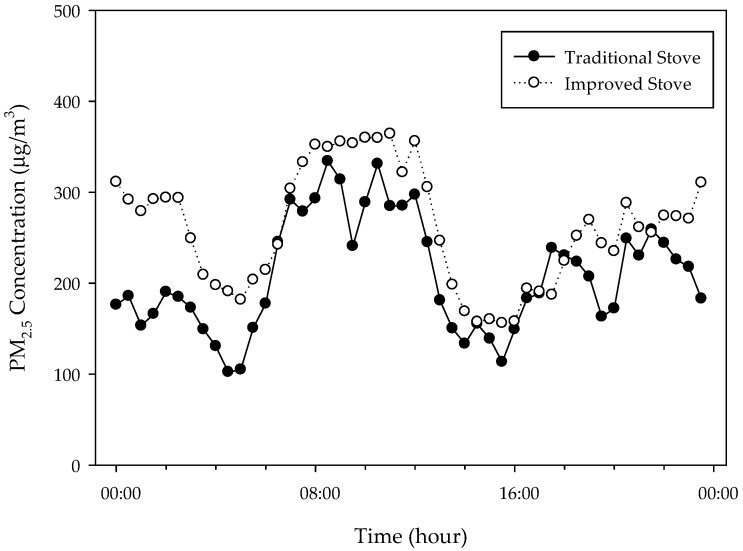
The 30 min average PM_2.5_ concentrations (μg/m^3^) in 60 gers according to stove type.

Figure 3 shows the 24 h temporal profiles of PM_2.5_ in gers with traditional and improved stoves. The indoor concentrations of PM_2.5_ started to increase at 6 a.m. and high concentrations were maintained in the morning. The concentration decreased continuously from 1 to 4 p.m., increased again beginning at about 4 p.m., and remained high until after midnight, when it decreased slowly until dawn. The highest and lowest 30 min average concentrations of the 60 gers were 344.8 μg/m^3^ at 10:30 a.m. and 135.9 μg/m^3^ at 3:30 p.m., respectively. Gers with improved stove tended to have higher 30 min average concentrations than those with traditional stove at most times. The average difference between the 24 h average PM_2.5_ concentrations in gers with traditional and improved stoves was 53.6 μg/m^3^, although the difference exceeded 100 μg/m^3^ from 23:30 p.m. until 2:30 a.m.

**Figure 4:**
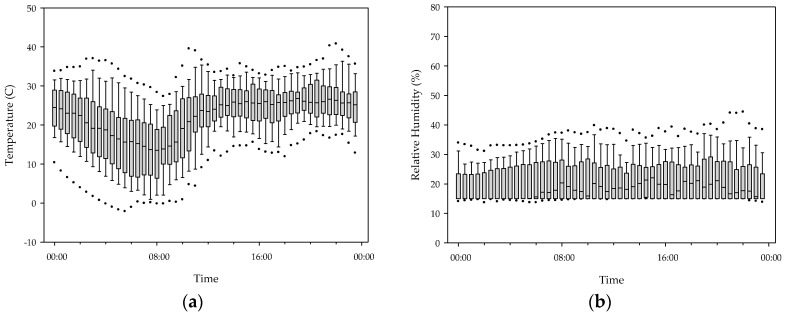
(**a**) Temperature and (**b**) relative humidity in 55 gers; outliers are beyond the 5th and 95th percentiles.

Figure 4 shows the 24 h temperature and relative humidity profiles in 55 gers. The indoor temperature increased continuously from 8:00 a.m. to 2:00 p.m. and remained at approximately 25 °C until 11:00 p.m., and then decreased during the night. The highest and lowest 30 min average temperatures were 26.9 °C at 10:00 p.m. and 13.4 °C at 8:00 a.m., respectively. The temperature variation was large and the maximum temperature was about twice the minimum temperature. Unlike the temperature, there was no temporal variation in relative humidity. The stove type did not affect the indoor temperature, while the relative humidity was always higher in gers with traditional stoves.

### 3.4. Temporal Profile of the Peak Frequency and Magnitude of PM_2.5_

The peak occurrence rate and peak magnitude were higher in gers with improved stove than in those with traditional stove. There were 15.0 ± 6.3 peaks per day in gers with traditional stove and 18.3 ± 7.2 per day in those with improved stove. The average peak magnitudes were 441.4 ± 246.0 and 458.3 ± 220.3 μg/m^3^, respectively. However, there were no statistical differences in either the peak occurrence rate (*p* = 0.062) or peak magnitude (*p* = 0.261).

**Figure 5:**
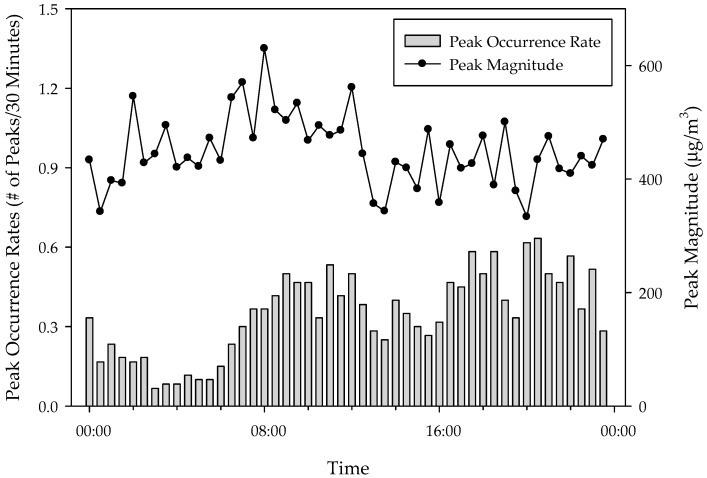
Peak occurrence rate (# of peaks/30 min) and peak magnitude (μg/m^3^) in 60 gers.

Figure 5 shows the temporal profiles of the peak occurrence rate (# of peaks/30 min) and peak magnitude (μg/m^3^) in 60 gers. The peak occurrence rate increased at 6:00 a.m. and again at 6:00 p.m. after a slight downturn in the afternoon. More peaks were observed in the evening, while fewer peaks were observed during the night. The peak occurrence rate profile was similar to the 24 h profile of PM_2.__5_ concentrations. Unlike the peak occurrence rate, the peak magnitude did not change over the 24 h.

## 4. Discussion

It was necessary to convert the PNC measured using the Dylos DC1700 into a mass concentration. Because light-scattering methods are affected by the characteristics of PM, it was necessary to calibrate the low-cost real-time monitoring device for different conditions. The calibration equation in this study of Dylos PNC-filter-weighted MicroPEM was obtained from 1st measurement during daytime in 24 gers. Since the 2nd measurement used only Dylos as the measuring device, it was not possible to obtain the conversion factor for each site. The PNC values of Dylos had to be converted using the calibration equation derived from the 1st measurement. Although there was limitation in did not calculate conversion factor at each measurement site, the single calibration equation could be applied because the indoor environment and main pollution source was similar in the gers.

This study derived a calibration equation between the Dylos and MicroPEM devices by linear regression for an indoor environment using coal as solid fuel (R^2^ = 0.68). Previous Dylos-PM_2.5_ calibration equations were derived from second hand smoking [17,18] or specific microenvironment [19,20]. In order to generate calibration equation of two devices, the linear equation was mainly used [18,19,21,22] and the quadratic equation [17] or the exponential equation [20] also used. The correlation of our study was relatively weak compare to R^2^ values of 0.86 [17] and 0.70–090 [19]. Also, the level of correlation coefficients was similar for both linear equation considering intercept (R^2^ = 0.69) and quadratic equation (R^2^ = 0.70). This R^2^ value might be associated with many measurement points at high concentrations above 100 μg/m^3^, since the uncertainties in PM concentrations were large at high concentration [20]. Nevertheless, low-cost real-time PM monitor could be useful tool for indoor pollution research in developing countries such as Mongolia. The equation derived from this study could be used to study specific pollution source, such as indoor coal combustion.

The indoor PM_2.5_ levels in the gers were very high and comparable to other studies of indoor environments where solid fuels are used. The geometric mean PM_2.5_ concentrations in homes burning smoky coal in Xuanwei (*n* = 122) and Fuyuan (*n* = 88), China, were 149 (GSD 2.0) and 138 (GSD 1.9) μg/m^3^, respectively [23]. In India, the 24 h PM_2.5_ concentrations in the kitchen area were 741 ± 539 μg/m^3^ in households using dung as a cooking fuel (*n* = 59) and 590 ± 575 in households using wood (*n* = 262) [24]. In both studies, the stove was located in a separate space, such as a kitchen or outside, while the stove in Mongolia is placed in the center of the ger. In this study, the 24 h average PM_2.5_ concentration of 60 households in gers was 9.4 times higher than the World Health Organization (WHO) air quality guideline of 25 μg/m^3^. Although the WHO air quality guidelines were developed for ambient air, they can be applied to indoor environments, specifically in the develop­ing world, where many people are exposed to high levels of combustion particles from indoor stoves and fires [25].

The indoor concentrations of PM_2.5_ were significantly higher in gers with improved stove than in those with traditional stove. Despite the similar fuel consumption and indoor temperature, the temporal profile indicated higher indoor PM_2.5_ concentrations in gers with improved stove. The PM_2.5_ concentration was higher in the indoor smoking gers (*n* = 7) than the non-smoking gers (*n* = 53), although the difference was not statistically significant (*p* = 0.061). The PM_2.5_ concentration of gers with improved stove was significantly higher, with or without including indoor smoking gers to the comparative groups. There was a significant difference only in the relative humidity depending on the stove type. The relative humidity was significantly higher in gers with traditional stove (23.3 ± 8.8%) than improved (18.6 ± 5.1%). When indoor PM_2.5_ concentrations were adjusted by the outdoor concentration, the I/O ratio was higher in gers with improved stove, although the difference by stove type was not statistically significant (*p* = 0.198). Both gers with traditional and improved stoves had an I/O ratio higher than 1.0 which indicates the significant presence of indoor particulate sources. It was difficult to obtain the I/O ratio for each ger directly because the winter temperature of Ulaanbaatar is extremely low and deviated from proper working temperature range of device. Therefore, the single point of nearest to the measurement area was used for outside air concentration value. Improved stoves were developed to reduce fuel consumption and pollutant emissions. However, the emissions efficiency during stove design was tested using the chimney method [26]. Any emissions tests should involve field tests to examine how residents are exposed while using the stoves.

Other factors affecting indoor air pollution were the structure and combustion method of the stoves. The traditional stoves with a low height are designed so that users first ignite wood and other kindling and then add coal on top of the burning kindling. In a traditional stove, fuel is added without extinguishing the fire. By contrast, the improved TLUD stoves were designed so that users place coal in the stove first and then start the combustion [13]. The improved stoves were designed to retain heat longer and require two ‘cold start’ fueling events per day, using less coal [26]. Combustion at low temperatures releases large amounts of PM. The high PM_2.5_ concentrations in gers with improved stove might be related to these design features and human behavior. A relatively dry indoor environment in the gers using improved stove might be associated with higher PM_2.5_ concentration.

The indoor temperatures in the gers were very high. During the monitoring period, the minimum outdoor temperature by month was −32 °C to −26.8 °C [27]. However, the indoor temperature averaged 22.1 ± 8.5 °C. The American Society of Heating, Refrigerating, and Air-Conditioning Engineers (ASHARE) recommends a comfortable range of acceptable humidity and operative temperature in indoor environment less than 0.2 m/s air speed and wearing cloth of 1.0 clo (1 clo = 0.155 m^2^ °C/W = 0.18 m^2^ h °C/kcal) in winter [28]. When the mean radiant temperature (MRT) due to indoor floor heating was considered, the comfort operative temperature was 17–21 °C in winter at 20% of relative humidity, which was similar to the inside of the gers. The temperature in the gers in winter was slightly higher than the comfort level. This suggests that excessive amounts of coal are used in the gers. Education programs may help reduce the use of coal in gers and maintain slightly lower indoor temperatures.

This study used peak analyses to account for the temporal variation in the indoor concentrations of PM_2.5_. A peak occurrence implied that there was an event during which the concentration of the pollutant sharply increased. It was necessary to identify the cause of such peaks because the number of peaks describes the PM_2.5_ concentration trend more accurately than the peak magnitude. Many factors can cause a peak, such as the amount of coal consumed, the type of coal burned, the number of times the stove is opened, and cooking with the stove. Smoking indoors may also affect the occurrence of peaks. According to observations made in previous studies [6,7], the residents of gers usually put coal in the stove at dawn to increase the indoor temperature and prepare breakfast. This was related to the increase in the PM_2.5_ concentration and peak occurrence rates around 6:00 a.m. However, we did not collect detailed information on the residents’ behaviors for quantitative analyses.

The most significant limitation of this study was the difficulty of sampling in gers. Random sampling in the ger district was not possible and no information was available regarding the statistical representativeness of population. The samples were recruited only by stove type without any prior knowledge of their characteristics. Because the two groups had similar characteristics, except relative humidity, the findings might not have been significantly influenced by the sampling strategy. Our findings suggest that the international support program has not effectively reduced indoor air quality in gers. This phenomenon was also found in a previous study [14]. Although our study might not have sufficient strength to confirm these findings, it warrants a larger scale study to evaluate the impact of the improved stoves on indoor air quality.

## 5. Conclusions

This study measured the 24 h residential indoor air quality of gers using a low-cost PM monitor and thermal environmental conditions. The PM_2.5_ concentration in the gers during winter exceeded the recommended level, and the gers with improved stove supported by international development programs did not reduce indoor PM_2.5_ concentrations. Also, the temperature in the gers was slightly higher than the indoor temperature recommended in winter. Better control measures for indoor coal combustion are needed to reduce the indoor concentrations of PM_2.5_ in gers.

## Figures and Tables

**Table 1:** Characteristics of the gers (*n* = 60).

Characteristic	Traditional Stove (*n* = 29)	Improved Stove (*n* = 31)	*p*-Value
Fuel type; Mixed fuels	29 (100%)	30 (96.4%)	-
Coal only	0 (0%)	1 (3.4%)	-
Amount of fuel (kg/day)	13.1 ± 7.5	11.9 ± 5.6	0.681
Frequency of adding fuel (event/day)	3.5 ± 1.7	3.3 ± 1.9	0.846
Use for cooking (event/day)	2.0 ± 1.0	2.4 ± 1.7	0.433
Number of family member	4.0 ± 1.8	4.0 ± 1.4	0.539
Smoking inside the ger	3 (10.3%)	4 (12.9%)	-
Temperature (°C) ^1^	22.3 ± 8.9	21.8 ± 8.1	0.105
Relative humidity (%) ^1^	23.3 ± 8.8	18.6 ± 5.1	0.000
PM_2.5_ concentration (μg/m^3^)	203.9 ± 195.1	257.5 ± 204.4	0.000
Indoor/Outdoor ratio of PM_2.5_	1.20 ± 0.68	1.50 ± 0.91	0.198

^1^ traditional stove *n* = 28, improved stove *n* = 27, total *n* = 55.
